# Surgical treatment of primitive gastro-intestinal lymphomas: a systematic review

**DOI:** 10.1186/1477-7819-9-145

**Published:** 2011-11-07

**Authors:** Roberto Cirocchi, Eriberto Farinella, Stefano Trastulli, Davide Cavaliere, Piero Covarelli, Chiara Listorti, Jacopo Desiderio, Francesco Barberini, Nicola Avenia, Antonio Rulli, Giorgio Maria Verdecchia, Giuseppe Noya, Carlo Boselli

**Affiliations:** 1 Department of Surgery, University of Perugia, Italy; 2 Department of Surgery, Charing Cross Hospital, Imperial College Healthcare NHS Foundation Trust, London, UK; 3 Unit of Surgical Oncology, Morgagni-Pierantoni Hospital, Forlì, Italy

## Abstract

Primitive Gastrointestinal Lymphomas (PGIL) are uncommon tumours, although time-trend analyses have demonstrated an increase. The role of surgery in the management of lymphoproliferative diseases has changed over the past 40 years. Nowadays their management is centred on systemic treatments as chemo-/radio- therapy. Surgery is restricted to very selected indications, always discussed in a multidisciplinary setting. The aim of this systematic review is to evaluate the actual role of surgery in the treatment of PGIL.

A systematic review of literature was conducted according to the recommendations of The Cochrane Collaboration. Main outcomes analysed were overall survival (OS) and disease free survival (DFS).

There are currently 1 RCT and 4 non-randomised prospective controlled studies comparing surgical versus medical treatment for PGIL. Seven hundred and one patients were analysed, divided into two groups: 318 who underwent to surgery alone or associated with chemotherapy and/or radiotherapy (surgical group) versus 383 who were treated with chemotherapy and/or radiotherapy (medical group).

Despite the OS at 10 years between surgical and medical groups did not show relevant differences, the DFS was significantly better in the medical group (P = 0.00001). Accordingly a trend was noticed in the recurrence rate, which was lower in the medical group (6.06 vs. 8.57%); and an higher mortality was revealed in the surgical group (4.51% vs. 1.50%).

The chemotherapy confirms its primary role in the management of PGIL as part of systemic treatment in the medical group. Surgery remains the treatment of choice in case of PGIL acutely complicated, although there is no evidence in literature regarding the utility of preventive surgery.

## Introduction

Primitive Gastrointestinal Lymphomas (PGIL) are uncommon tumours, although time-trend analyses have demonstrated an increase of 2.7% per annum in incidence for gastric (6.3%) and small bowel diseases (5.9%) [1].

PGIL could be localised in any site of the gastrointestinal tract [1-7]. The most frequent site is the stomach (44-75%). Other locations might be the jejunum or the ileo-cecal region, while duodenum, colon and rectum are rare. Multiple gastrointestinal lesions are very infrequent.

The treatment of patient with PGIL is quite undefined. In fact, although the efficacy of chemotherapy (CT) is well recognised and all treatment strategies for PGIL include CT, with or without radiotherapy (RT); whether or not CT should be performed as unique medical treatment or as part of a combined treatment, which includes the surgical resection of the primary lymphoma, is still discussed. Moreover, surgery is sometime necessary to manage acute complications, such as haemorrhage, abscess, gastrointestinal occlusion or perforation during systemic therapies or suggested for prevention of such emergencies.

The aim of this systematic review is to evaluate the actual role of surgery in the treatment of PGIL, analysing overall and disease free survival as main outcomes.

## Methods of meta-analysis

We conducted the review according to the recommendations of The Cochrane Collaboration and performed the statistical analysis using Review Manager 5 (RevMan) software.

### Research methods for identification of studies

We searched for all published and unpublished randomised controlled trials (RCT) and controlled clinical trials (CCT) using the following electronic databases: Cochrane Central Register of Controlled Trials, MEDLINE, Science Citation Index, ISI Proceedings, Current Controlled Trials metaRegister, Zetoc, CINAHL and EMBASE. The following medical search headings (MeSH) and free text words were used: ''surgery''; "chemotherapy"; "radiotherapy"; "gastric lymphoma"; ''gastrointestinal lymphoma", "colonic lymphoma". We checked the reference lists of all relevant studies obtained from our search and from previously published systematic reviews in order to identify other possible articles. The latest date for this search was February 25^th ^2010.

### Data Extraction

Three authors (RC and ST) assessed titles or abstracts of all the studies identified by the initial search and excluded clearly non-relevant studies. They obtained the full text of all potentially relevant studies and also those with unclear methodology. These studies were assessed by the authors as to whether they met the inclusion criteria for this review. Disagreements on inclusion were resolved by discussing and, if necessary, by involving an independent third author (EF).

### Inclusion Criteria

To be included in the analysis, the studies had to compare surgery alone or associated with chemotherapy and/or radiotherapy (surgical group) versus chemotherapy and/or radiotherapy (medical group) in the treatment of gastrointestinal lymphoma tumours.

### Exclusion Criteria

Studies were excluded from the meta-analysis if the outcomes of interest were not reported for both groups, or solid tumours were considered, or there was a considerable overlap between authors, centres or patient cohorts evaluated.

### Outcomes of Interest

Primary outcomes analysed were: overall survival (OS) and disease free survival (DFS). Secondary outcomes measured were: recurrence rate and mortality.

### Measures of treatment effect

Statistical analysis for categorical variables was performed by using the odds ratio (OR). This ratio represents the odds of an adverse event occurring in the surgical treatment group compared with the medical treatment group. The Mantel-Haenszel method was used to combine the ORs for the outcomes of interest. Intention-to-treat analyses were performed extracting the number of patients originally allocated to each treatment group irrespective of compliance. Results were presented on a forest plot graphs.

### Assessment of heterogeneity

Heterogeneity was first tested using Chi-squared test. A Chi-squared test with a P value < 0.100 representing statistical significance. However, since tests of heterogeneity had a relative low power when there were few study we further explored heterogeneity derived from another statistical method named "inconsistency" or I^2 ^metric, which is independent of the number of combined studies. If I^2 ^is equal 0%, there is no heterogeneity. If I^2 ^> 50% heterogeneity is indicated.

## Results for the meta-analysis

### Eligible Studies

**Figure 1:**
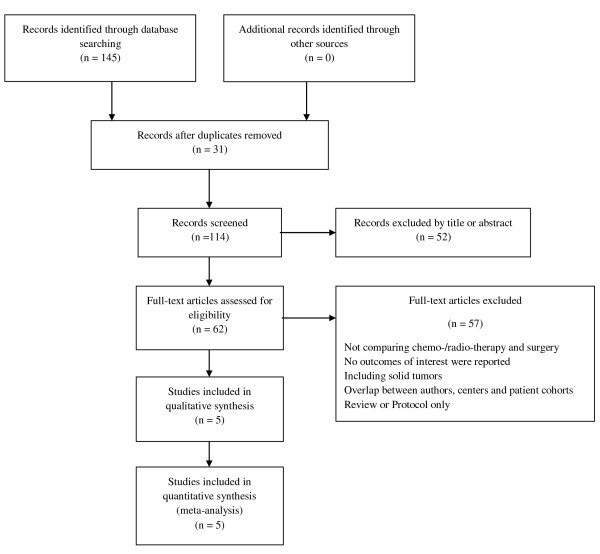
**Study selection flow chart**.

Using the search strategy listed above, 114 publications were identified. Fifty-two studies were excluded following title and abstract review. The remaining 62 studies were investigated in detail and 57 studies were excluded as they did not meet the inclusion criteria for this review (Figure 1).

There were 1 RCT and 4 non-randomised prospective controlled studies comparing surgical versus medical treatment for PGIL, which fulfilled the inclusion criteria and were analysed in the systematic review (Table 1) [6,8-11]. Seven hundred and one patients were analysed, divided into two groups: 318 who underwent to surgery alone or associated with chemotherapy and/or radiotherapy (surgical group) versus 383 who were treated with chemotherapy and/or radiotherapy (medical group). Only in the randomised trial [8] the patients were divided in three different group: surgery (80 patients), radiotherapy (78 patients) and chemotherapy (83 patients).

**Table 1:** Characteristics of the included studies

Author/Year	Types of study	N° of total evaluated patients	Inclusion criteria	Surgical treatment +/- medical theraphy	Medical theraphy alone	Mean follow-up	Results	
							
							Overall survival	Event-free survival
Avilés et al. [8] 2005	RCT Open-label	241	Patients with low-grade gastric MALT lymphoma age < 70 yr old, no gender difference, ECOG status ≤2, immunodeficiency virus test negative, tumor mass > 5, previously untreated, stage I or IIE (according to the Lugano Conference criteria)	80 patients received surgery alone (total gastrectomy)	78 patients received only radiotherapy 83 patients received only chemotherapy	7, 5 years (range 4.8-11.6 yr)	10 years 80% S group 75% R group 85% C group	10 years 52% S group 52% R group 87% C group

Gobbi et al. [10] 2000	PNR	154	Patients who fulfilled Lewin's criteria for diagnosing PGL (stomach and intestinal). Low-grade MALT lymphomas were excluded from this study	106 patients received chemotherapy plus surgery	48 patients received chemotherapy Radiotherapy was optionally given only when residual tumor masses seemed to persist at restaging after primary therapy or when bulky masses were present at onset.	NI	NI	NI

Popescu et al. [9] 1990	PNR	37	Patients with a histological diagnosis of intermediate or high-grade NHL according to the Working Formulation (WF) involving the stomach were included. Patients who received radiotherapy but no chemotherapy treatment were not included. Patients in whom lymphoma diagnosis predated demonstration of gastric involvement or where the bulk of the disease and its manifestations was extra-abdominal, nodal, hepatic or splenic were considered to have secondary involvement of the stomach were excluded.	13 Surgery and chemotherapy 5 total gastrectomy 8 partial gastrectomy	24 patients received chemotherapy alone	53 months	5 years 60% in S+C group 67% in medical therapy group	5 years 85 > % % in S+C group 62% in medical therapy group

Binn et al. [11] 2003	PNR Multicentric	84	Patients with diffuse large B-cell primary gastric lymphoma with stage IE and IIE according to the Ann Arbor staging system. Mediterranean lymphoma, human immunodeficiency virus-related lymphoma and post-transplantation lymphoma were not included.	40 patients received surgery plus chemotherapy 21 total gastrectomy 19 partial gastrectomy	44 patients received chemotherapy alone 7 patients received additional radiotherapy	59 months (range 3-128)	5 years 90, 5% in S+C group 91, 1% in medical therapy group	5 years 85, 5% in S+C group 91, 6% in medical terapy group

Koch et al. [6] 2001	PNR Multicentric	185	Patients with all histological tips of gastric low and high grade lymphoma but only in stage I E and II E 1- 2. Patients who were older than 75 years and/or presented with second malignancies, had missing confirmation of histologic subtype by central review, or had comorbidity prohibiting therapy were excluded from study	79 patients received complete or partial resection in combination with radio- and/or chemotherapy	106 patients received only radio- and/or chemotherapy	52 months (range 0-92 months)	5 years 84, 2% in medical therapy group 82% Combined surgical treatment	5 years 78.7% in medical therapy group 78, 9% Combined surgical treatment

RCT = randomised clinical trial; PNR = prospective non randomised; NI = not indicated; S = surgery; R = radiotherapy; C = chemotherapy

### Results of Meta-analysis

**Figure 2:**
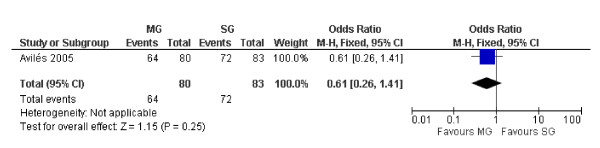
**The overall survival at 10 years in patients with PGIL treated with chemo and/or radiotherapy versus the surgical approach associated with adjuvant treatments**. Surgical Group (SG): surgery alone or associated with chemotherapy and/or radiotherapy. Medical Group (MG): chemotherapy and/or radiotherapy.

**Figure 3:**
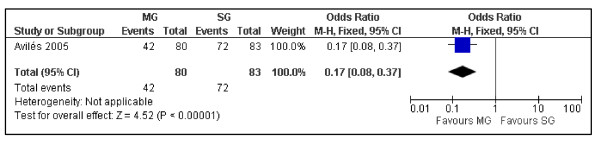
**The disease free survival at 10 years in patients with PGIL treated with chemo and/or radiotherapy versus the surgical approach associated with adjuvant treatments**. Surgical Group (SG): surgery alone or associated with chemotherapy and/or radiotherapy. Medical Group (MG): chemotherapy and/or radiotherapy.

**Figure 4:**
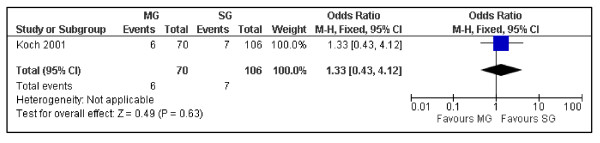
**The incidence of recurrences in patients with PGIL treated with chemo and/or radiotherapy versus the surgical approach associated with adjuvant treatments**. Surgical Group (SG): surgery alone or associated with chemotherapy and/or radiotherapy. Medical Group (MG): chemotherapy and/or radiotherapy.

**Figure 5:**
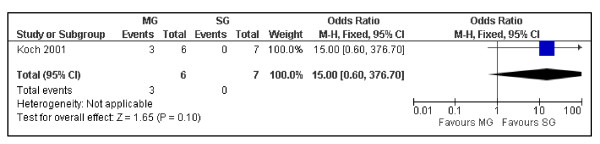
**The mortality in patients with recurrence from PGIL treated with chemo and/or radiotherapy versus the surgical approach associated with adjuvant treatments**. Surgical Group (SG): surgery alone or associated with chemotherapy and/or radiotherapy. Medical Group (MG): chemotherapy and/or radiotherapy.

**Figure 6:**
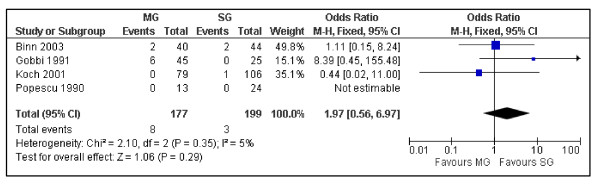
**The mortality in patients with PGIL treated with chemo and/or radiotherapy versus the surgical approach associated with adjuvant treatments**. Surgical Group (SG): surgery alone or associated with chemotherapy and/or radiotherapy. Medical Group (MG): chemotherapy and/or radiotherapy.

Despite the OS at 10 years between surgical and medical groups did not show relevant differences (P = 0.25) (Figure 2), the DFS was significantly better in the medical group (P = 0.00001) (Figure 3). Despite not statistically significant, a trend was noticed in the recurrence rate, which was lower in the medical group (6.06 vs. 8.57%) (P = 0.63) (Figure 4). Furthermore the recurrences after surgical treatment were associated with higher mortality (50% vs. 0%) (P = 0.10) (Figure 5). Similarly an higher mortality was revealed in the surgical group (4.51% vs. 1.50%) (P = 0.29) (Figure 6).

## Discussion

Based on the assumption that PGIL is a localised disease, the surgical treatment was traditionally considered the cornerstone of the therapeutical strategy showing impressive results in terms of long DFS and OS [3,12-16].

Nowadays this approach has been extensively revised and the management of PGIL is centred on systemic treatments such as chemo- and radiotherapy.

The current National Comprehensive Cancer Network (NCCN) guidelines [17,18] suggests for the gastric MALT lymphoma chemotherapy mainly and Helicobacter pylori eradication therapy in the early stage. Surgery is restricted to the treatment of complications, such as occlusion, bleeding or perforation. Preventive surgery is sometime advocated in bulky tumours, when rapid tumour necrosis secondary to chemo-/radiotherapy may be associated with a high risk of life threatening complications. Surgery is also required for removal of residual disease after medical debulking [19]. Total gastrectomy is the most frequent procedure performed for gastric MALT lymphomas, given the evidence that they are multicentric; a D2 lymphadenectomy is recommended [20].

The majority of small bowel lymphomas are represented by B-large cell lymphomas. The NCCN guidelines proposes surgery or radiotherapy as equally effective in the early stage of MALT lymphomas, while chemotherapy for B-large cell lymphomas and advanced stage of MALT lymphomas. In locally advanced lymphomas of the small bowel, surgical resection is indicated during laparotomy/laparoscopy for tumours of undefined histology or complicated by intestinal occlusion, bleeding, and perforation. Surgery may be advocated before chemotherapy in bulky lesions in order to prevent bowel perforation. A segmental intestinal resection with the own mesentery containing at least 12 lymph nodes is recommended.

In the colon-rectum localization, the MALT lymphomas are more common. The NCCN proposed for the colon the same protocols as for the small intestine. In this cases the surgical approach is represented by the segmental resection of the colon, or a local excision for rectal tumours.

The MALT lymphomas represent the majority of PGIL [21], therefore the disease stage is commonly IE [6] with a favourable prognosis [21].

Given the actual dominant role of chemotherapy in the treatment of PGIL, in our literature research most of outcomes resulted from combined therapy. We could identify only one trial [8] analysing surgery, radiotherapy and chemotherapy separately. In this trial, Aviles et al included only patients with diagnosis of low-grade gastric MALT lymphoma, who were randomised to be treated with primary surgical resection (total gastrectomy and D2 limphadenectomy), radiotherapy or chemotherapy. At 4 weeks complete response was achieved in all patients, but relapse in another abdominal site were more frequent in patients treated with surgery or radiotherapy. At 10 years DFS and OS were statistical significantly higher in the chemotherapy group (p = 0.01 and p = 0.04).

Surgery lost its leading role, becoming the treatment of choice only in acute complicated cases or in the prevention of chemotherapy and/or radiotherapy related complications secondary to rapid tumour necrosis [22]. The aim of preventive surgery is to reduce the high incidence of severe morbidity and mortality due to an emergency laparotomy in highly compromised patients [23]. In the past this risk was overestimated and a surgical management was more frequently advocated; actually it stands at 5% [24]: surgery has more than 5% of procedure related morbidity [25] and similarly, from our meta-analysis resulted a higher mortality (P = 0, 29). Therefore surgery must be reserved to very selected patients.

One of the main limitations of our study is the retrospective nature of the majority of studies included in the systematic review. These studies are heterogeneous, combining different types of malignant lymphoma, using different histology classifications and staging systems. Moreover, the aim of this review was the comparison of surgery versus medical therapies but only one study confronted these two approaches. In the others studies, surgery was part of a multimodal treatment, associated to chemo with or without radiotherapy. Besides, case history considers different type of lymphomas, in different stages, with different prognosis, without stratification. Therefore, the application of selective methods and statistical analysis, even if apparently they are in line with what is the generally accepted, they cannot bring to evidence based conclusions.

It would be interesting to analyze only studies including surgery during not surgical treatments in order to evaluate if, when and why surgery was used. From this type of analysis prognostic factors for development of acute complications could be evident and could help selecting high risk patients that are preemptively candidate for surgery.

## Conclusions

Although from our meta-analysis there was not any significant difference in terms of OS between surgical and medical groups, DFS was significantly better in the medical group. Accordingly a lower recurrence rate was reported in the medical group. Moreover, our meta-analysis showed an higher mortality in the surgical group. This confirms the widely recognized primary role of the chemotherapy, as part of systemic treatment in the medical group. Surgery remains the treatment of choice in case of PGIL acutely complicated, although there is no evidence in literature regarding the utility of preventive surgery.

Despite the absence in literature of high quality studies (RCT) demonstrating the effectiveness of chemotherapy without local surgical resection in patient with PGIL, the evidence present in literature and analyzed in our review well support a systemic approach for PGIL patients.

## Competing interests

The authors declare that they have no competing interests.

## Authors' contributions

* All authors contributed equally to this work and approved the final manuscript.

## References

[B1] Gurney KA Cartwright RA Gilman EA Descriptive epidemiology of gastrointestinal non-Hodgkin's lymphoma in a population-based registry Br J Cancer 1999 79 11-12 1929 34 10206316 10.1038/sj.bjc.6690307 PMC2362786

[B2] Freeman C Berg JW Cutler SJ Occurrence and prognosis of extranodal lymphomas Cancer 1972 29 1 252 60 10.1002/1097-0142(197201)29:1<252::AID-CNCR2820290138>3.0.CO;2-# 5007387

[B3] Radaszkiewicz T Dragosics B Bauer P Gastrointestinal malignant lymphomas of the mucosa-associated lymphoid tissue: factors relevant to prognosis Gastroenterology 1992 102 5 1628 38 1568573 10.1016/0016-5085(92)91723-h

[B4] d'Amore F Brincker H Gronbaek K Thorling K Pedersen M Jensen MK Mortensen LS Non-Hodgkin's lymphoma of the gastrointestinal tract: a population-based analysis of incidence, geographic distribution, clinicopathologic presentation features, and prognosis. Danish Lymphoma Study Group J Clin Oncol 1994 12 8 1673 84 8040680 10.1200/JCO.1994.12.8.1673

[B5] Liang R Todd D Chan TK Chiu E Lie A Kwong YL Choy D Ho FC Prognostic factors for primary gastrointestinal lymphoma Hematol Oncol 1995 13 3 153 63 10.1002/hon.2900130305 7622145

[B6] Koch P del Valle F Berdel WE Willich NA Reers B Hiddemann W Grothaus-Pinke B Reinartz G Brockmann J Temmesfeld A Schmitz R Rübe C Probst A Jaenke G Bodenstein H Junker A Pott C Schultze J Heinecke A Parwaresch R Tiemann M Primary gastrointestinal non-Hodgkin's lymphoma: II: Combined surgical and conservative or conservative management only in localized gastric lymphoma--results of the prospective German Multicenter Study GIT NHL 01/92 J Clin Oncol 2001 19 18 3874 83 11559725 10.1200/JCO.2001.19.18.3874

[B7] Nakamura S Matsumoto T Iida M Yao T Tsuneyoshi M Primary gastrointestinal lymphoma in Japan: a clinicopathologic analysis of 455 patients with special reference to its time trends Cancer 2003 97 10 2462 73 10.1002/cncr.11415 12733145

[B8] Aviles A Nambo MJ Neri N Talavera A Cleto S Mucosa-associated lymphoid tissue (MALT) lymphoma of the stomach: results of a controlled clinical trial Med Oncol 2005 22 1 57 62 10.1385/MO:22:1:057 15750197

[B9] Popescu RA Wotherspoon AC Cunningham D Norman A Prendiville J Hill ME Surgery plus chemotherapy or chemotherapy alone for primary intermediate- and high-grade gastric non-Hodgkin's lymphoma: the Royal Marsden Hospital experience Eur J Cancer 1999 35 6 928 34 10.1016/S0959-8049(99)00069-6 10533473

[B10] Gobbi PG Ghirardelli ML Cavalli C Baldini L Broglia C Clo V Bertè R Ilariucci F Carotenuto M Piccinini L Stelitano C Attardo-Parrinello G Ascari E The role of surgery in the treatment of gastrointestinal lymphomas other than low-grade MALT lymphomas Haematologica 2000 85 4 372 80 10756362

[B11] Binn M Ruskone-Fourmestraux A Lepage E Haioun C Delmer A Aegerter P Lavergne A Guettier C Delchier JC Surgical resection plus chemotherapy versus chemotherapy alone: comparison of two strategies to treat diffuse large B-cell gastric lymphoma Ann Oncol 2003 14 12 1751 7 10.1093/annonc/mdg495 14630680

[B12] Cogliatti SB Schmid U Schumacher U Eckert F Hansmann ML Hedderich J Takahashi H Lennert K Primary B-cell gastric lymphoma: a clinicopathological study of 145 patients Gastroenterology 1991 101 5 1159 70 1936785 10.1016/0016-5085(91)90063-q

[B13] Seifert E Schulte F Stolte M Long-term results of treatment of malignant non-Hodgkin's lymphoma of the stomach Z Gastroenterol 1992 30 8 505 8 1413932

[B14] Montalban C Castrillo JM Abraira V Serrano M Bellas C Piris MA Gastric B-cell mucosa-associated lymphoid tissue (MALT) lymphoma. Clinicopathological study and evaluation of the prognostic factors in 143 patients Ann Oncol 1995 6 4 355 62 7619750 10.1093/oxfordjournals.annonc.a059184

[B15] Pasini F Ambrosetti A Sabbioni R Todeschini G Santo A Meneghini V Perona G Cetto GL Postoperative chemotherapy increases the disease-free survival rate in primary gastric lymphomas stage IE and IIE Eur J Cancer 1994 30A 1 33 6 7511402 10.1016/s0959-8049(05)80014-0

[B16] Bartlett DL Karpeh MS Filippa DA Brennan MF Long-term follow-up after curative surgery for early gastric lymphoma Ann Surg 1996 223 1 53 62 10.1097/00000658-199601000-00008 8554419 PMC1235063

[B17] National Comprehensive Cancer Network http://www.nccn.org/professionals/physician_gls/PDF/nhl.pdf

[B18] Zucca E Cavalli F Gut lymphomas Baillieres Clin Haematol 1996 9 4 727 41 10.1016/S0950-3536(96)80051-5 9138615

[B19] Yoon SS Coit DG Portlock CS Karpeh MS The diminishing role of surgery in the treatment of gastric lymphoma Ann Surg 2004 240 1 28 37 10.1097/01.sla.0000129356.81281.0c 15213615 PMC1356371

[B20] Kodera Y Yamamura Y Nakamura S Shimizu Y Torii A Hirai T Yasui K Morimoto T Kato T Kito T The role of radical gastrectomy with systematic lymphadenectomy for the diagnosis and treatment of primary gastric lymphoma Ann Surg 1998 227 1 45 50 10.1097/00000658-199801000-00007 9445109 PMC1191171

[B21] Day D Jass J Price A Shepherd N JM S Non-epithelial tumours of the stomach 2003 Malden (USA): Blackwell Publishing Inc.

[B22] Aviles A Nambo MJ Neri N Huerta-Guzman J Cuadra I Alvarado I Castañeda C Fernández R González M The role of surgery in primary gastric lymphoma: results of a controlled clinical trial Ann Surg 2004 240 1 44 50 10.1097/01.sla.0000129354.31318.f1 15213617 PMC1356373

[B23] Al-Refaie W Abdalla E Ahmad S Mansfield P Gastric cancer 2006 Houston (USA) Lippincott Williams & Wilkins

[B24] Mercer DW Robinson EK editors Gastric neoplasia 2007 Philadelphia

[B25] Friedberg J Mauch P Rimsza L Fisher R editors 2008 Non-Hodgkin's Lymphomas: Lippincott Williams & Wilkins

